# Genetic Algorithm with Maximum-Minimum Crossover (GA-MMC) Applied in Optimization of Radiation Pattern Control of Phased-Array Radars for Rocket Tracking Systems

**DOI:** 10.3390/s140815113

**Published:** 2014-08-18

**Authors:** Leonardo W. T. Silva, Vitor F. Barros, Sandro G. Silva

**Affiliations:** 1 Launching Center of Barreira do Inferno, Brazilian Air Force, RN-063 59140-970, Parnamirim RN, Brazil; 2 Department of Electrical Engineering, Federal University of Rio Grande do Norte, Campus Universitário 59078-900, Natal RN, Brazil; E-Mail: vfb3916@yahoo.com.br; 3 Department of Communications Engineering, Federal University of Rio Grande do Norte, Campus Universitário 59078-900, Natal RN, Brazil; E-Mail: sgsee@ufrnet.br

**Keywords:** rocket tracking systems, phased array radars, radiation pattern control, genetic algorithm, maximum-minimum crossover

## Abstract

In launching operations, Rocket Tracking Systems (RTS) process the trajectory data obtained by radar sensors. In order to improve functionality and maintenance, radars can be upgraded by replacing antennas with parabolic reflectors (PRs) with phased arrays (PAs). These arrays enable the electronic control of the radiation pattern by adjusting the signal supplied to each radiating element. However, in projects of phased array radars (PARs), the modeling of the problem is subject to various combinations of excitation signals producing a complex optimization problem. In this case, it is possible to calculate the problem solutions with optimization methods such as genetic algorithms (GAs). For this, the Genetic Algorithm with Maximum-Minimum Crossover (GA-MMC) method was developed to control the radiation pattern of PAs. The GA-MMC uses a reconfigurable algorithm with multiple objectives, differentiated coding and a new crossover genetic operator. This operator has a different approach from the conventional one, because it performs the crossover of the fittest individuals with the least fit individuals in order to enhance the genetic diversity. Thus, GA-MMC was successful in more than 90% of the tests for each application, increased the fitness of the final population by more than 20% and reduced the premature convergence.

## 1. Introduction

Nowadays, aerospace technology and knowledge are part of everyday life in civil and military applications, being used, for example, at weather stations, aircrafts, weapon systems and satellite networks. In many of these applications, large and long range aerospace vehicles (e.g., rockets) are used for spatial surveys, air defense or satellite transportation. These features are found in launching centers with appropriate locations and specialized infrastructure to execute scientific and commercial activities [1–6]. The activities of these centers can be divided into two areas: launching and tracking. The first one is responsible for preparing and integrating vehicles and their payloads, besides doing the launching itself from platforms. On the other hand, the tracking area obtains in real time the location of the launched vehicles and their likely point of impact, for monitoring and safety purposes.

The main element of the tracking area is the Rocket Tracking System (RTS), which consists of radar sensors and is responsible for collecting and processing trajectory data. Parabolic reflector radars (PRRs) are usually used, due to their long range and high pointing accuracy [3,7,8]. However, there are Phased Arrays Radars (PARs), which consist of antenna arrays that allow electronic control of the radiation pattern, eliminating electromechanical devices [9,10]. Therewith, new functionalities are offered, since it is now possible to determine the shape of the radiation pattern and to increase the scanning speed. At the same time, reliability is increased by removing electromechanical devices, maintenance is reduced and it is possible to continue tracking even when the sensor is degraded, that is, if there is damage to some of the array elements during tracking.

Arrays directly used as transmitting and/or receiving antennas are well known in the control of the radiation pattern [11]. Transmitarrays are another option in which the arrays are set as lenses that shape the radiation pattern after being traversed by antenna-radiated waves [12]. In both cases, adjustment of the radiation pattern in real time requires electronic control with an active array, whose excitation can be adjusted in each element in order to regulate their radiation characteristics. The electronic control is dynamic, less susceptible to failures and has become more common with the increasing capacity and decreasing costs of digital processors. However, due to the large number of possible combinations for the excitation signals of the array elements, the electronic control should be carefully designed, aided by optimization methods.

Thus, the PARs radiation pattern control for an RTS relies on three steps: specification, modeling and implementation. The specification is determined by requirements of the application and the implementation is determined by the available technology and associated costs, so that the greatest difficulty for the designer lies in the modeling step, due to an optimization problem with various boundary conditions imposed by the other steps. Such optimization is a difficult task, which demands the designer to have experience and time. In order to ease such process, flexible optimization tools have been used, which can be applied in different situations and requirements, at the expense of small adjustments. An example occurs in PARs, when they are initially designed for a particular application but, over time, end up having new functions, so that it is necessary to redo the setting of the radiation pattern of a phased array (PA) already built, in order to meet new operating requirements.

The aforementioned flexibility is achieved through the probabilistic optimization method of genetic algorithms (GAs), which allows converging to a set of optimal or nearly optimal solutions through the application of Darwin's Theory of Evolution and fundamentals of genetics [13–19]. They manipulate the numerous restrictions imposed on projects in several areas, with a fairly generic method. So, the GAs are quite useful in optimizing the design of PAs, which is a nonlinear problem of multiple objectives, in which conventional deterministic methods are vulnerable to a local optimum [13,16,20,21–23]. Thus, the GA can calculate the optimal values of excitation signals of the array elements, which will be used to control the radiation pattern in several applications, such as directing the main beam, determining the width and the number of beams, increasing gain and scanning speed, reducing sidelobes, rejecting interfering signals or improving the coverage area of the radiated signal.

In this context, the Genetic Algorithm with Maximum-Minimum Crossover (GA-MMC) optimization method was developed to control the radiation pattern of PAs. The GA-MMC uses a reconfigurable algorithm with multiple objectives, and has two special features. The first is the real coding with a differentiated technique, inspired by the physical structure of the antenna array itself, in order to simplify the algorithm and reduce the processing time. Another remarkable feature of GA is the new crossover genetic operator, called Maximum-Minimum Crossover (MMC), which combines the most and least fit individuals to increase the genetic diversity of the population and, consequently, the global search, improving the final fitness of the solution set and reducing the processing time.

To test the GA-MMC validity, a linear antenna array was adopted, because it serves as the basis for composing more complex arrays and has proven practical utility (as in radars with rotating base or cell phone towers). It also represents a more challenging task for the optimization method, since it has less freedom to format the radiation pattern compared to a planar array. This requires a more precise control of the elements excitation signals, in order to achieve extreme operating conditions, such as main beam near endfire direction. Moreover, as the GA-MMC is generic and allows adaptation to any type of element, isotropic radiators were chosen, which can be easily extended to other types of elements, such as dipoles or microstrip antennas [7].

With that said, this paper is organized as follows: in Section 2, PARs are described, considering RTS operation and the theory of antenna arrays. In Section 3, GA-MMC is presented, using GA concepts in optimization problems in controlling the radiation pattern of PAs. In Section 4, the simulation results are shown and discussed. Finally, the paper is concluded in Section 5.

## 2. Phased Array Radar (PAR)

### 2.1. Rocket Tracking System (RTS)

#### 2.1.1. Features, Requirements and Functions

**Figure 1.**
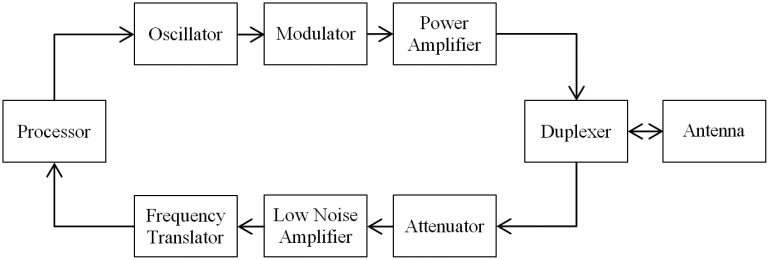
Block diagram of a tracking radar.

RTS is the main component of the tracking area and consists of radar sensors that collect and process data from the target trajectory. However, despite the current knowledge about radars, the construction of an RTS involves significant technical challenges because the target may have a reduced cross section and be hundreds of miles away, which leads to the need for high power transmitters (peak values of hundreds of kW) and antennas with fast scanning and high precision. Figure 1 shows a block diagram of a tracking radar.

The devices that perform the tasks of each block must be designed so as to meet various operational radar requirements, such as accurate tracking in three-dimensional position, long distances range, high reliability, easy maintenance, low probability of false alarms, detection at extreme conditions (stealth targets or those with very high or very low speeds), clutter suppression, noise filtering in RF circuits and interference inhibition caused by commercial transmitters or countermeasures (e.g., jammers) [9].

Moreover, in the most difficult operating conditions, both processor and antenna deserve special attention. The antennas will be examined in Section 2.2. As for the processors, there has been great progress in technology of digital signals through the high integration of semiconductors, miniaturized microwave integrated circuits and computational-aided designs for microwave circuits. Signal and data processing must extract information from the target, such as format, position and speed, as well as cancel clutter, noise and interference, which could saturate the receiver, preventing detection of real targets or generating false target detection [11,24].

Data filtering is a part of this process, which removes data generated by error detection and calculates future position data in order to direct antennas. In this case, the tracking success depends on selecting the appropriate dynamic models of target movement [25,26], considering that targets in general, such as commercial aircrafts, rockets or missiles, can be characterized by three basic dynamic movements: constant speed motion, accelerated motion and rotational motion [27]. A dynamic model widely used is the Kalman filter, which is the basis for the development of many others models [28].

Thus, the main measurement of radar systems, both for tracking and surveillance, are the three-dimensional position coordinates of the target (*x*, *y* and *z*, in Cartesian coordinates). The basic difference is that tracking seeks to detect and pursue a specific target, while surveillance monitors a region of space to detect unknown targets [29]. Hence, when tracking, in addition to position detection, the sensor must provide a quick and accurate estimation of future kinematic parameters of the target [30]. Thereby, considering the scanning time, the tracking processing must be much faster (around milliseconds) than the surveillance one (about 5 s [25]).

Apart from processing, the functions of tracking and surveillance differ in terms of radar-target distance and response speed of the radar. In general, during surveillance, as in air traffic control, the target is moving with its main axis approximately parallel to the velocity vector and is far from the radar [31], so it is reasonable to assume that the target and radar are in the same horizontal plane [32,33]. However, in tracking, this assumption cannot be considered, especially for the so-called platform or pad radars. These radar sensors are positioned near the launching site, aiming to track rockets since ignition time, so they are subject to quick changes in elevation angle, which requires a high response speed to the radar beam motion.

**Figure 2.**
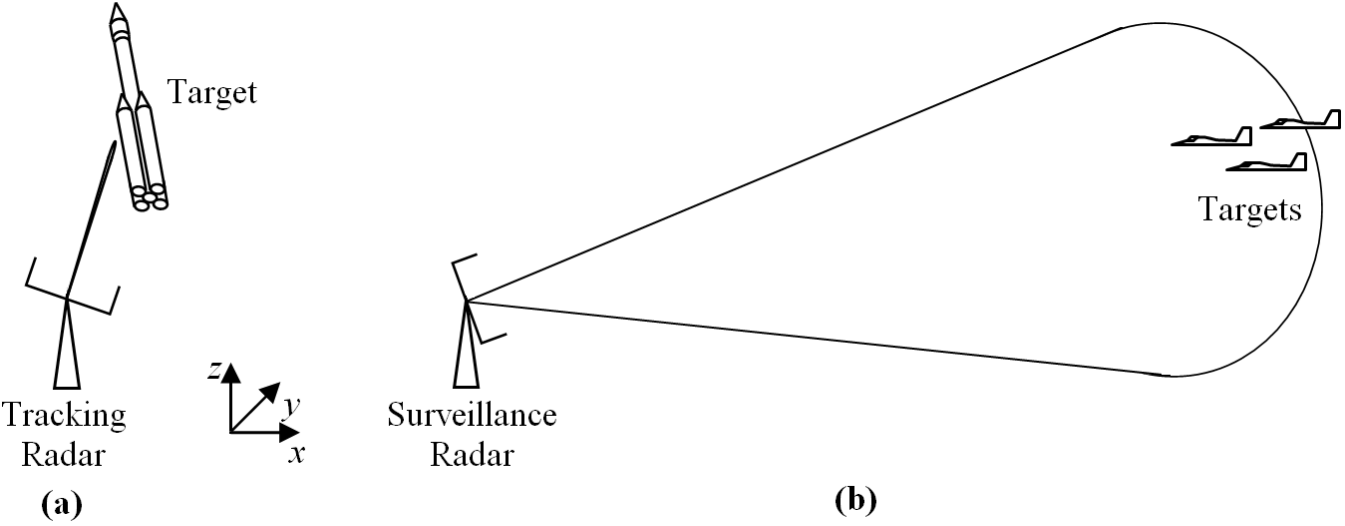
(**a**) Rocket tracking radar near launch pad. (**b**) Surveillance radar for remote control air traffic.

In normal operation, another difference is the beamwidth. Generally, due to accuracy requirements to measure the target position, the beamwidth of tracking radars is often very narrow (for example, the order of 1.0°–2.5° [3,28,34]), while in surveillance radars, which seek to sweep a large area at one time, beams tend to be large (for example, range from 15°–90° [34,35]). Despite these differences, it is possible to obtain a multifunctional system with hybrid radar sensors to perform simultaneously tasks of tracking and surveillance [29]. To do so, taking into account the design and implementation complexity, it is possible to use PARs, which are more flexible. Hence, these multifunctional systems can be obtained from tracking systems, by making the necessary changes in the scanning process of radar sensors to also perform surveillance tasks, such as air defense and missile warning. In Figure 2, an illustration is shown to compare the differences in beamwidth and radar-target distance between a tracking radar and a surveillance radar.

#### 2.1.2. RTS Operation

To determine the trajectory of a target, the RTS must go through some steps. Initially, one must determine the tracking mode, which can be either radar or transponder. The transponder mode allows greater range and works with different frequencies of transmission and reception, since the signal received by the radar sensor is the “response” sent by the transponder installed on the target. On the radar mode (also called skin tracking), the range is smaller and the frequency is the same for both transmission and reception, since the received signal is an echo of the signal transmitted by the radar, and it is reflected by the target. In transponder mode, tests are conducted to check if the target is in communication with the radar. Tracking is in automatic mode since the moment of launch, with the radar correcting its position without relying on manual intervention. However, if the tracking is on radar mode, it is necessary to know the expected trajectory of the target, called the nominal trajectory (NT), so that the radar operator directs the antenna to certain locals (holding points) of the NT, in order to locate the target in space and start automatic tracking.

If, during the flight, an abnormality occurs and the radar loses automatic tracking, there are some alternatives to resume tracking. First, simpler and less accurate, is manual scanning, in which the operator positions the radar antenna near where location was lost or in the next holding point of the NT. This option is less recommended, due to the high speed at which the target (e.g., rocket or missile) changes position. The second alternative is called designation, which is obtained from data from other radar sensors or NT itself.

The designation originates from the existing redundancy in the network of radar sensors that integrate an RTS, so that, preferably, more than one radar is tracking the target at the same time. This is possible if each radar sensor operates at a different frequency, thus avoiding interference among them. However, different frequencies must be previously established and properly controlled, including the fidelity of the transmission equipment, in order to ensure that the pre-set values will be accurate and will not interfere with each other, such as the production of harmonics or unwanted noise in each radar sensor transmitter.

By ensuring this separation into different frequencies, it is possible to build an RTS network [36], which enables tracking of vehicles over long trajectories, the so-called tracking network. This is possible because the radar sensors of the network are interconnected and strategically placed in different locations, so that each radar tracks the target during part of its trajectory. This could not be done by radars near the platform, due to lack of visibility, either by obstacles in the direct sight line or the curvature of Earth itself. Thus, radar sensors have target visibility only for a limited period of flight and when they are close to losing target location, they inform the next network radar, which resumes tracking.

The data obtained by the network allow a more robust performance [37,38] and a more precise estimate of the target position [39]. This happens because radars operate with redundancy, preventing the loss of location by one radar harming the tracking as a whole. Moreover, it enables the comparison of data from multiple radars (during or after tracking) to check the reliability and accuracy of the measurement. Therefore, redundancy in an RTS prevents errors caused by human failures, equipment breakdowns, unexpected behaviors of targets (such as abrupt maneuvers or fragmentation) and undesirable conditions in the propagation environment (such as lack of direct sight line, clutter, noise and interference).

**Figure 3.**
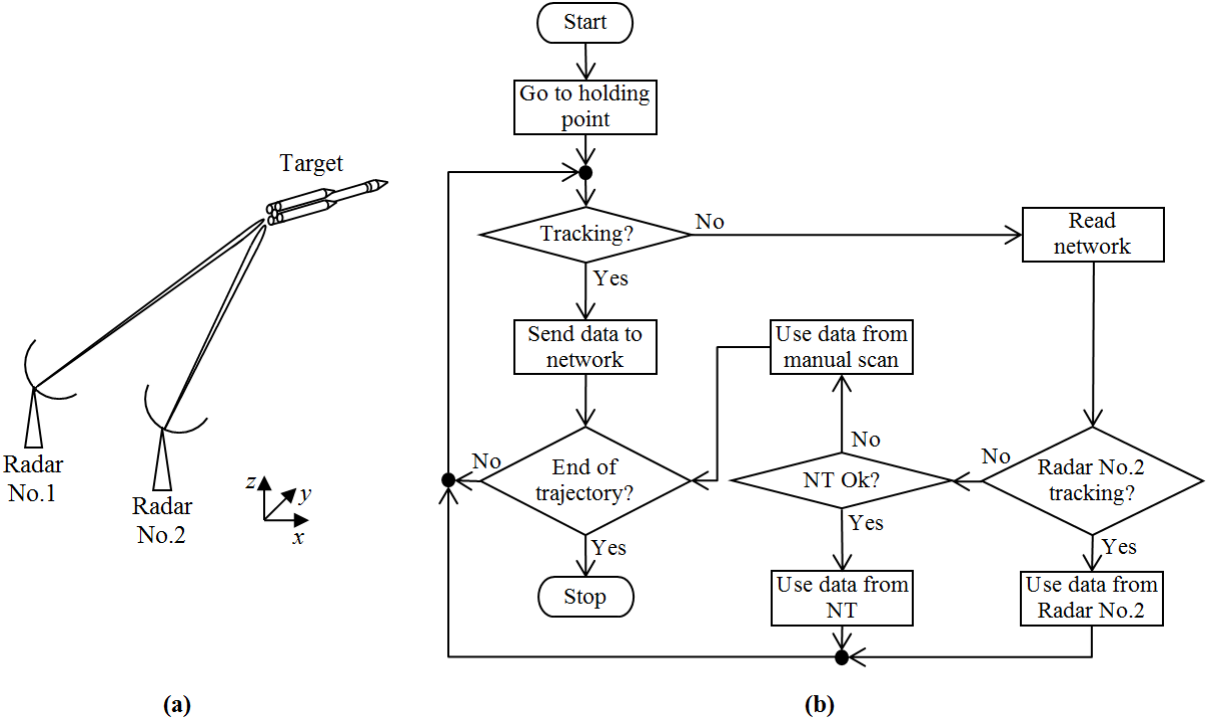
(**a**) Rocket Tracking System (RTS) with two radar sensors. (**b**) Flowchart of operation of the Radar No.1.

Thus, if a radar sensor misses the target location, some other radar's information on the network is used as a backup to locate the target again. Before being transferred between different radar sensors, the information must be processed, in order to correct the delay time and differences in the origin of coordinates systems. On the other hand, if no radar is tracking, it is possible to opt for the designation of NT, which is far more accurate than manual scanning. However, NT is not completely reliable since climatic conditions (such as wind direction and speed) and changes in the target behavior during propulsion might significantly change the real trajectory in relation to the NT. In Figure 3, there is an RTS with two radar sensors in network and a flowchart of Radar No. 1 operation.

### 2.2. Radiation Pattern Control

#### 2.2.1. Phased Array (PA)

Radiation characteristics of the antenna must meet the design requirements, and they vary according to its shape and building materials, which are factors that determine the distribution of electric and magnetic fields. Typically, for a single antenna, the distribution of these fields, seen by the radiation pattern, is relatively sparse and has low values of gain [7]. However, applications such as RTS radar sensors require antennas with high gain and control, which can be made by combining several radiator elements in a single structure, called antenna array [7,8,20]. In the array, besides the geometry of elements (spatial distribution and distance) and the type of antenna (wires, loops, apertures, microstrip or a mix of them), it is possible to dynamically format the radiation pattern by controlling the amplitude and phase of excitation signals (electronic control) of each element, originating PAs [7].

Nowadays, with increasingly faster and cheaper digital processors, such control has become more common, enabling new functionalities, such as improving coverage area, quality of signal transmitted/received (greater gain) and interference suppression. This is achieved by factors like an accurate positioning of the main beam tilt, beamwidth and beam number flexibility, high scanning speeds, increased gain, reduced sidelobes and rejection of interfering signals (null positioning).

**Figure 4.**
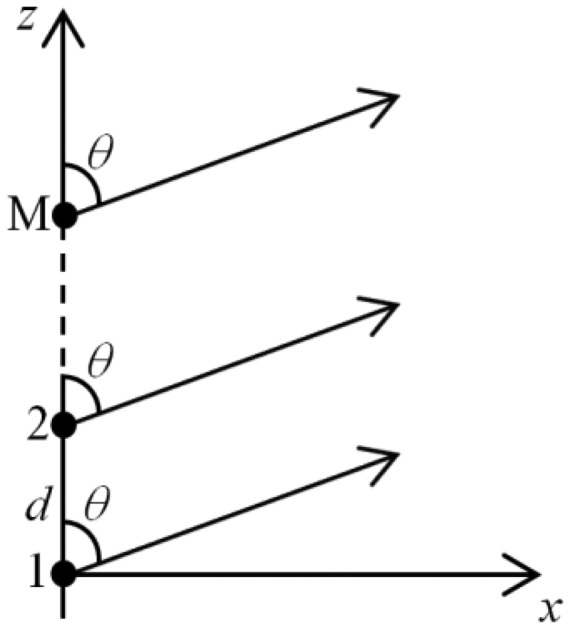
Linear array of M isotropic elements.

To build PAs, elements are distributed along an axis (linear array), a flat surface (planar array) or 3D surface (three dimensional array). Neglecting the mutual coupling among the elements, the total fields of PA are obtained by a vector composition among fields of their elements. Therefore, high gain is achieved by the action of constructive interference in the desired direction and destructive interference in other directions. In Figure 4, there is a linear array of isotropic elements, often used to form more complex antennas. The planar array, for example, can be considered a “linear array of linear arrays”. Planar arrays require more complex excitation schemes, although they allow greater control of the radiation characteristics, such as a symmetrical radiation pattern and smaller sidelobes.

The PA total far field (*E_T_*) is equal to the field of an element positioned at the reference point (*E_el_*) multiplied by the array field, or array factor, *AF*, as summarized in Equation (1) [7]. This process is called multiplication of radiation patterns and can be extended to PAs of several elements [7,8]. In general, *AF* depends only on the geometry and excitation of PA, which eases projects designed to isotropic elements and then extrapolated to any type of element.

(1) $$E_{T}=E_{el}\cdot AF$$

The *AF* allows, then, to match PA radiation to the desired application with the tilt angle of the main lobe ranging between broadside and endfire directions. Besides that, it reduces the level of sidelobes, avoiding interference, regions without coverage and power loss in the main direction [7,13]. In this case, the radiation pattern can be traced from the radiation intensity, *U*, which for isotropic elements is equal to the square of *AF* [7]. For a linear array with variation only in tilt angle, θ, the normalized expression of *U*, in dB, is given in Equation (2). The parameter used to quantify the level of sidelobes is the Relative Side Lobe Level (RSLL), calculated as the ratio between the amplitudes of the main lobe, *A_M_*, and the greater sidelobe, *A_S_*, according to (3) [20].

(2) $$U_{dB}\left(\theta \right)=20\cdot \text{log}\left(\frac{AF}{\text{max}\left(AF\right)}\right)$$

(3) $$\text{RSLL}=200\cdot \text{log}\left(\frac{A_{M}}{A_{S}}\right)$$

The *AF* for a symmetrical linear array with an even number of elements (*N* = 2*M*) is given by Equation (4), being *A_n_* the excitation amplitudes, β the constant phase difference between adjacent elements (progressive phase), *d* the uniform spacing between elements, λ the wavelength and θ the angle of propagation related to the array axis [7].

(4) $$AF_{2M}\left(\theta \right)=\sum_{n=1}^{M}A_{n}\cdot \text{cos}\left[\left(2n-1\right)\cdot \left(\frac{\pi d}{\lambda }\cdot \text{cos}\theta +\beta \right)\right]$$

If compared to other techniques, electronic control has more flexibility, durability and functionality, being built by power dividers and phase shifters. It is common to use amplitude to control the level of sidelobes and to use phase to control the main lobe direction, so that the adopted configuration depends on a balance between gain, sidelobe level and beamwidth [7,13]. Some conventional deterministic methods for electronic control are the Uniform, Binomial and Dolph-Tschebyscheff, apart from probabilistic methods, such as GAs.

#### 2.2.2. Applications

RTS is usually used at launching and tracking centers with parabolic reflector radars (PRRs) [1–6], due to their long range and high pointing accuracy [7,8]. On the other hand, PRRs have some restrictions, such as (a) difficulty in locating the target in space, due to very narrow beam and high target speed after launch, (b) single beam presence, not allowing multiple tracking, like stage-separated vehicles, aircraft-launched missile testing (at the instant of aircraft-missile separation) or accidents with vehicle fragmentation, (c) rotations in antenna azimuth and elevation are made by electromechanical devices (servomechanisms), at low speed and with a greater need for maintenance, (d) breakdown in any antenna device may render the radar sensor inoperative.

To overcome such restrictions, an alternative is to employ an RTS with PARs, whose sensors are composed of antenna arrays to electronically control the radiation pattern [9,10]. This generates new functionalities, such as larger scanning areas at critical times (shortly after launch) and the possibility of tracking multiple targets simultaneously. Moreover, without electromechanical devices, there are less conversion errors, fewer losses and lower number of maintenance, which improves calibration, efficiency and reliability. It is also possible to track even if the sensor is deteriorated, in the case of damage to certain elements of the array during tracking.

Thus, PARs are used for surveillance and tracking from the 1960s up until nowadays, providing attack warnings by enemy missiles and monitoring objects in orbit. As examples, there are Ballistic Missile Early Warning System (BMEWS), Missile Site Radar (MSR), Cobra Dane (AN/FPS-108), PAVE PAWS (AN/FPS-115) and Cobra Judy (AN/SPQ-11), all developed by the U.S. Department of Defense [28,34].

Other examples of possible applications of PARs are: allocation of radiation pattern nulls in the direction of arrival of interfering signals, caused by harmonics in commercial transmitters or countermeasures (jammers) [11,40–44]; variation of the format of radiation pattern of radar sensors of a RTS, based on time multiplexing between wide beams (larger scanning area) and narrow beams (greater pointing accuracy) [29,44]; tracking each member of an aircraft formation, even if these members maneuver relatively close to one another, so that the formation does not confuse the defense system [45].

**Figure 5.**
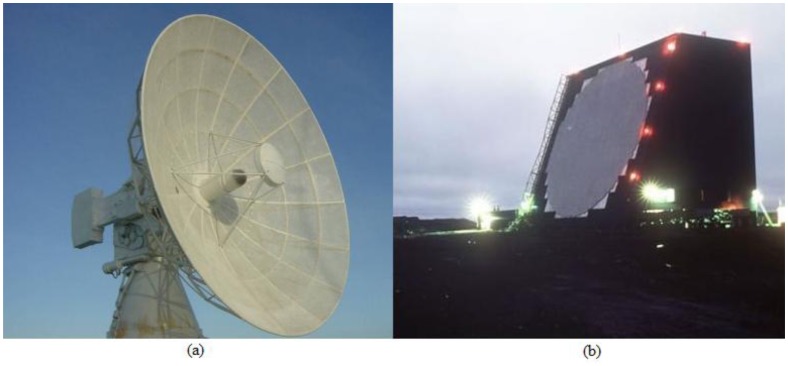
(**a**) Bearn radar [46]. (**b**) Cobra Dane radar [47].

To illustrate applications of PRRs and PARs, in Figure 5, there are: (a) Bearn radar, with parabolic reflector (PR) in the C band, used for rockets and missiles tracking, at Launching Center of Barreira do Inferno, Brazilian Air Force, in Parnamirim, RN, Brazil [46], (b) Cobra Dane Radar, PA in the L band, used for missile surveillance and subsequently monitoring space at Shemya Base, U.S. Air Force, Shemya Island, AK, USA [47].

**Figure 6.**
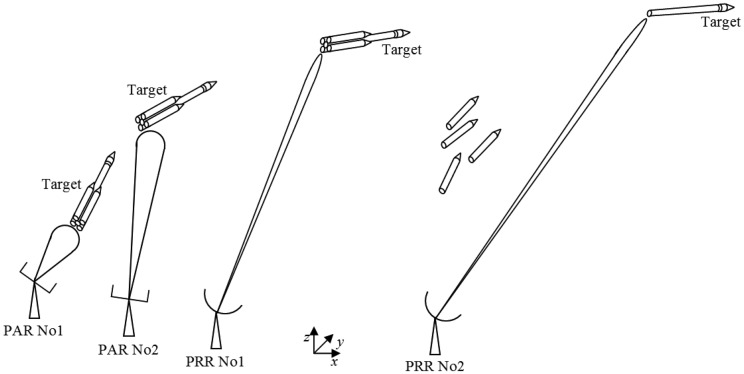
Tracking network with phased array radars (PARs) and parabolic reflector radars (PRRs).

On the other hand, the development of PAs does not mean disuse of PR conventional radars. An interesting option is to make a composition between these two radars, in order to take advantage of both settings. This is the case for the Active Phased Array Radar (APAR), developed by Thales Nederland (Hollandse Signaalapparaten Besloten Vennootschap) to accomplish air defense for German, Canadian and Dutch battleships, in which there is a combination between a PA radar and a long distance conventional surveillance system (SMART-L) [29]. In the RTS case, an association between PARs and PRRs can be performed. The PARs would be responsible for the tracking soon after launching, acting as platform radars, due to their wider and faster beams, for the period in which the rocket speed is high, and the risks of losing the target location are higher. On the other hand, conventional PRRs would take on tracking for longer trajectories, acting as distance radars, since they have high power and narrow beams. This association between PARs and PRRs is shown in Figure 6.

#### 2.2.3. Implementation

Although the use of PAs in radar systems has been consolidated for quite some time, their construction can be rather complex, requiring many elements and connections, depending on the application requirements, such as the main beam pointing accuracy, reducing sidelobes or nulls level of the radiation pattern [35]. Therefore, the electronic control of the radiation pattern has proven to be one of the main challenges in the design of antennas for civil and military radar systems [12,15,48]. The PAs designer analyzes details of the array and constraints imposed by the radar system to try and optimize the design, whereas the analysis of a PA is much more complex than that of a passive antenna positioned mechanically, due to variation of performance characteristics in the array according to the scanning angle [10]. Also, in order to control radiation pattern, PAs are composed of several elements fed with variable amplitude and phase, or through a time delay in each element excitation [10,35].

Determining weights of amplitude and phase of PA elements can be made by several offline or online (real time) optimization methods with various kinds of adaptive algorithms for controlling the radiation pattern [25,28,36,44,49]. One must highlight the results of offline calculation of sidelobe suppression from Electronic Sidelobes Steerable Radar (ELRA) [41]. In offline mode, when a pointing angle is specified, there is a previously calculated table by the algorithm with the combination of signals corresponding to that angle [48]. Once in real time, there is no such table and the algorithm is executed online during the tracking. It is common for PAs to possess many elements, such as: Hard Point Demonstration Array Radar (HAPDAR) with 4300 elements [35], Multi-function Electronically Scanned Adaptive Radar (MESAR) with 900 elements [40], and ELRA with 300 elements on the transmitter and another 768 elements on the receiver [41]. Nevertheless, a possible difficulty for PAs is the limited search region, since, as a general rule, the maximum scanning angle of a planar array is 60° in broadside, which limits the face scanning of the planar array to 120° [9,41].

**Figure 7.**
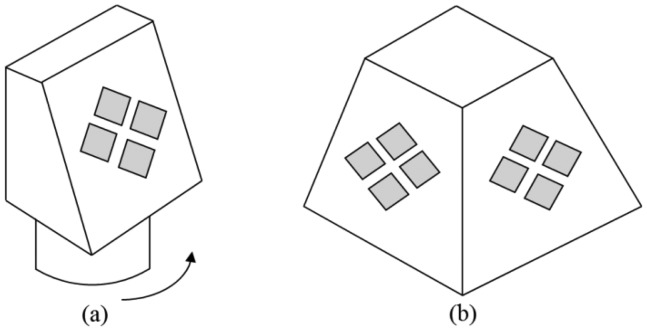
Scanning alternatives. (**a**) Rotational basis. (**b**) Pyramidal basis.

Thus, alternatives are necessary for a complete azimuth scan. The first alternative, used on fighter aircrafts, is a 3D array conformed to the fuselage. Its elements are active in all directions at the same time, providing a wider visual field and a greater scanning speed. Such implementation, however, can be quite costly [9]. Another alternative is a planar array on a mechanical rotating base, which would be a cheaper solution, despite inherently bringing the disadvantages of electromechanical devices, such as lower scanning speed and higher possibility of defects [43]. An intermediate alternative is to use four planar arrays, forming the faces of a pyramid, in order to achieve the best balance between cost and scanned area. This solution was built by the United States for air defense, both on ground and ships [42]. In Figure 7, there is an outline of the two last mentioned solutions.

## 3. Genetic Algorithm with Maximum-Minimum Crossover (GA-MMC)

### 3.1. Optimization of Radiation Pattern Control of PAs

#### 3.1.1. Optimization Problem

Among the three steps related to controlling radiation pattern of phased array radars (PARs) for a rocket tracking system (RTS), the greatest difficulty for the designer resides in the modeling, because it involves an optimization problem with various boundary conditions, imposed by the other steps (specification and implementation). Such optimization is a difficult task, which demands experience and time, so it needs the aid of computational tools that can be applied in different situations and requirements at the expense of small adaptations. One example occurs with PARs used in new applications. They require the adjustment of the PA radiation pattern for the new operation settings.

Therefore, the design of PAs is a complex problem because it uses arrays with large numbers of elements and various combinations of excitation signals producing a large number of possible solutions. Additionally, beyond conventional optimization objectives, such as main lobe scanning and sidelobe reduction, it has been common to reject interfering signals due to applications in electronic warfare and eliminating interference caused by commercial activities near the PAR.

In this context, there are several deterministic and probabilistic optimization methods to calculate solutions for that problem. In simpler cases, where only one or two optimization objectives were needed, conventional deterministic methods are very popular. Among them, the Dolph-Tschebyscheff method stands out because it is an intermediate solution between the Uniform and Binomial methods [7,50]. However, the deterministic methods may fail when there is nonlinearity, discontinuity, multimodality, multiple optimization objectives and vast spaces of search, or noises [13,16,17].

When deterministic methods do not apply, probabilistic methods are used, such as genetic algorithms (GAs), which explore large search spaces and manipulate a large number of constraints. Therefore, GAs are remarkable in design optimization of PAs, since this is a nonlinear problem of multiple objectives, in which conventional deterministic methods are vulnerable to local optimum [13,16,18–23,51–59]. Thus, despite the amount of calculation, at the end of the process, GAs outperform results achieved by conventional methods [54].

#### 3.1.2. Genetic Algorithms (GAs)

GAs are quite useful in optimizing the design of PAs, as they optimize the calculation of excitation signals values of the array elements, which will be used for online control of the radiation pattern in several applications. In general, GA is a computational search method, which allows to converge to a set of optimal or quasi-optimal solutions through the application of Darwin's Theory of Evolution and fundamentals of genetics [13–19]. In GAs, a population of possible solutions to the problem evolves according to probabilistic operators designed from biological metaphors, so there is a tendency that, on average, the individuals become increasingly better solutions as the evolutionary process goes on [16]. Therefore, GAs can be said as optimization methods for the solution of problems through “survival of the fittest” [60–62]. It has become a popular type of evolutionary computation, whose innovation is in a stochastic model that uses a population of solutions instead of a single one [63]. The evolutionary computing techniques are excellent for complex problems, for they are able to find better results in shorter periods of time [64].

In such problems, the optimization criterion involves choosing a set of variables to maximize (or minimize) a function of optimization (objective function), in order to explore search spaces of large dimensions, and manipulate a great number of requirements. In addition, GAs are applied to problems in all areas, because they are responsive to most problems, which may include: multiple optimization objectives; simple algorithms; capability of operating in discontinuous and non-differentiable search spaces; absence of a starting point to begin searching; faster processing and the possibility of being associated to other techniques.

**Figure 8.**
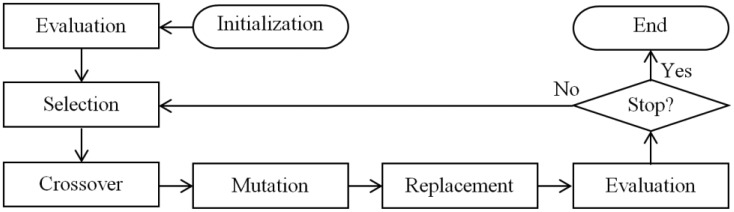
Flowchart of Genetic Algorithm with Maximum-Minimum Crossover (GA-MMC) [13].

As for their operation, GAs operate concurrently with a set of points (population of individuals), which represent a search space of coded solutions [13]. Complex mathematical expressions are not used, such as in the case of the traditional gradient method [17]. This avoids using differential calculus or specific knowledge of the problem. Instead, much simpler rules and operators are applied over a fitness value, which are obtained by objective function for each individual in the population. Due to their versatility, GAs do not have a rigid model, but usually have a similar implementation flow [13,14,16]. This flow starts by generating a set of possible solutions to the problem, all coded according to a predetermined rule of representation. This set is nothing more than the initial population, which is modified for each full cycle of iteration, called generation. Individuals with higher fitness values have a greater chance to survive. They are also more likely to be selected as “parents” to generate a new individual [63,65,66]. In the flowchart of Figure 8, there is an example of GA, which is used in the GA-MMC [13].

The performance of a GA depends heavily on the parameters used (population size and rates of crossover and mutation), which are crucial to finding an optimal solution to the problem [65]. The choice of parameters may lead the evolution to a quick halt, with the population getting stagnant at a local optimum value, and thus does not meet the requirements for the problem. To avoid such premature convergence, it is common to increase the value of mutation genetic operator, in order to raise the genetic diversity of the population. On other hand, it can turn the algorithm in an almost random search. As for the optimum values, as with any optimization with multiple objectives, there is no single optimal solution, but a set of equally optimal solutions (multimodality). One of them should be chosen according to the requirements of application. Thus, to evaluate the optimization provided by the GA, one must compare the fittest individual with the intended objective. If this objective is not previously known, or is not reached after countless generations, the comparison can be made with a randomly extracted solution from the population, or by an average of the population as a whole [63].

Such a comparison is necessary to adopt a stopping criterion for the GA, since the processing time is an important factor, introducing a trade-off between the accuracy required and the time available for optimization [66]. That is, in multimodal problems, it is preferable to obtain a sub-optimal solution in a feasible time rather than wandering in search of the absolute optimal solution. As GAs have several probabilistic operators that use random responses, the path towards the optimal solution is not always the same, and the study of the convergence characteristics should consider the average or most of the results of several tests [22]. In addition, many bio-inspired simulation-based studies, such as GAs, may have its performance verified by real scenarios [65]. For instance, the optimal solution can be implemented in hardware using circuit techniques [66]. This makes it possible to check the validity of the performed optimization. However, such work should consider the costs involved as well as its real need, since many problems may already have consolidated technological solutions, requiring only experimental testing.

### 3.2. Structure of Genetic Algorithm with Maximum-Minimum Crossover (GA-MMC)

#### 3.2.1. Introductory Remarks

The GA-MMC method aims at optimizing the control of the radiation pattern of PARs, whose features are multiple objectives, a differentiated real coding and new crossover genetic operator. Additionally, the GA-MMC has a modular format in order to make it generic and easily reconfigurable for other applications, requiring only minor changes to the input parameters and the objective function.

#### 3.2.2. Coding

**Figure 9.**
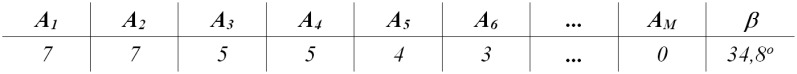
Individual with real coding used in GA-MMC [13].

The excitation of array elements is best represented by the real coding [51,54]. Compared to binary and Gray codes, the real coding is faster, eliminates the relationship between precision and number of bits, its results are easier to reproduce and it avoids changes of GA in each application. In GA-MCC, the PA is an individual with one chromosome of fixed size, whose genes are the amplitudes, *A_n_*, and the progressive phase, β, of the array elements excitation. The values that the genes can take are given by the allowed levels of excitation variation, and the search space is proportional to these levels. Thus, a different way of real coding was used, inspired by the physical structure of the PA itself, so that genes are naturally ordered to the direct application of genetic operators, simplifying the algorithm and reducing processing time. An individual example (0 ≤ *A_n_* ≤ 7 and β ≤ 180°) with the adopted real coding is illustrated in Figure 9.

#### 3.2.3. Initialization

The initial population of possible solutions (or individuals) is generated with the greatest possible genetic diversity to explore concurrently the search space which characterizes the advantage of GA over conventional methods. Populations tend to have fixed size, and values of 30–100 individuals usually resolve most problems [20,22,53]. Knowing that, GA-MMC uses a population of 100 individuals, randomly generated to increase genetic diversity.

The maximum and minimum values of *A_n_* and β are determined by the proposed application. In the tests, taking into account practical restrictions [20,53] eight excitation levels are used (*A_n_* varies from 0–7, with increment equal to 1) and 629 angles of phase (β varies from –π to +π, with increment equal to 0.01 rad). Even with these restrictions, for the linear array of 40 elements used in the tests, there is a wide search space with 7.252 × 10^20^ possible solutions, which confirms the choice of GA to optimize the problem.

#### 3.2.4. Evaluation

The quality of each individual in the population is considered by its fitness value, *f_i_*, obtained from the objective function, which uses a weighting between optimization objectives. GA-MMC was then tested in two applications. In the first one, the objectives are to control the tilt angle of the main lobe and to maximize the RSLL, with the objective function given by Equation (5), in which *A_M_* is the main lobe amplitude, *A_S_* is the greater sidelobe amplitude, θ*_C_* is the calculated tilt angle, θ_0_ is the desired tilt angle and *K_M_* is the main lobe adjustment factor [20]. The values *A_M_* and *A_S_* are obtained from *AF* in Equation (4), considering θ_0_ varying from 0 rad (0°) until π rad (180°) with resolution, Δθ, equal to 0.01 rad (0,573°), in a total of 315 scanning angles. Due to the importance of the tilt angle in PARs, the objective function prioritizes θ_0_. Hence, the term containing RSLL (*A_M_*/*A_S_*) just weighs the fitness value, while the θ_0_ is inserted into a non-linear term, producing a fitness peak to the desired angle, θ*_C_* = θ_0_, and a rapid reduction in fitness as it moves away from θ_0_. *K_M_* enables to control the influence of θ_0_ in the fitness value, and the algorithm tests indicated *K_M_* = 1:

(5) $$f_{i}=\frac{A_{M}}{A_{S}}\cdot \frac{1}{1+K_{M}\cdot {\left(\theta_{C}-\theta_{0}\right)}^{\text{2}}}$$

In the second application of GA-MMC, besides controlling θ_0_ and maximizing RSLL, the objective function aims at positioning nulls of the radiation pattern to reject two interfering signals (e.g., jammers), *A*_I1_ and *A*_I2_, according to Equation (6). The null value is given by the signal-to-interference ratio, *SIR*, between main lobe (*A_M_*) and interference (maximum value between *A*_I1_ and *A*_I2_). Null position in the directions of interferences is essential, so that the calculated *SIR* should be greater than or equal to the desired value. Therefore, the term involving *SIR* in Equation (6) is nonlinear, so that: (i) it does not alter fitness, if *A_M_*/max (*A*_I1_, *A*_I2_) ≥ *SIR*, (ii) it reduces fitness by 75% if *A_M_*/*A_I1_* ≤ *SIR* or *A_M_*/*A*_I2_ ≤ *SIR*, and (iii) it reduces fitness in 93.75% if *A_M_*/*A*_I1_ ≤ *SIR* and *A_M_*/*A*_I2_ ≤ *SIR*. Thus, θ_0_ has priority, as in Equation (5), because one must have θ*_C_* = θ_0_ to prevent fitness reduction. On the other hand, a *SIR* higher than desired does not alter the results, because, in current use, right positioning of the main beam is more important than positioning nulls towards the interferences direction.

(6) $$\begin{array}{c} f_{i}=\frac{A_{M}}{A_{S}}\cdot \frac{1}{1+K_{M}\cdot {\left(\theta_{T}-\theta_{0}\right)}^{2}}\cdot {\text{2}}^{\left(\frac{X}{abs\left(X\right)}-1\right)}\cdot {\text{2}}^{\left(\frac{Y}{abs\left(Y\right)}-1\right)}\\ X=\frac{A_{M}}{A_{\text{i1}}}-SIR\;\&\;Y=\frac{A_{M}}{A_{\text{i2}}}-SIR \end{array}$$

#### 3.2.5. Selection

The GA should select individuals in the population in order to generate more qualified descendants, but, at the same time, it should avoid that only the fittest individual be selected, preventing premature convergence in a local optimum. This selection can be executed by several deterministic or probabilistic methods, but it is always based on the fitness value. Because of the simple implementation and low computational complexity (depends on the size of the extracted set, *n*, and not on population size, *N*), GA-MMC adopts the technique of stochastic tournament, performing the probabilistic selection of a set with only two individuals in the population (*n* = 2).

In the traditional tournament mode, only the fittest individual of such pair is used for reproduction process [16]. However, on GA-MMC, changes in that process were made, aiming for the crossover to provide a greater genetic diversity. Thus, the selection of the first individual to reproduce uses the traditional model, whereas the second individual is chosen by the smallest fitness of the pair, originating the MMC method. Furthermore, selected individuals are always put back in the population, which enables them to be selected again, so the process is repeated until the number of individuals required for reproduction is reached.

#### 3.2.6. Maximum-Minimum Crossover (MMC)

The crossover genetic operator acts at the local search, extracting parts of two individuals (parents) and combining them to form the offspring (children), in order to get a better fit. Without this crossover, a random search takes place. By contrast, the union between selection and crossover performs a local search near the best solutions, being the GA main search tool [21]. In GA-MMC, the differentiated coding mode, inspired by the physical structure of PA (Figure 9), enables the crossover to make the direct exchange of genes from parents, using real numbers. Without the need to convert into binary system, such as in conventional GA, processing time is quite reduced.

**Figure 10.**
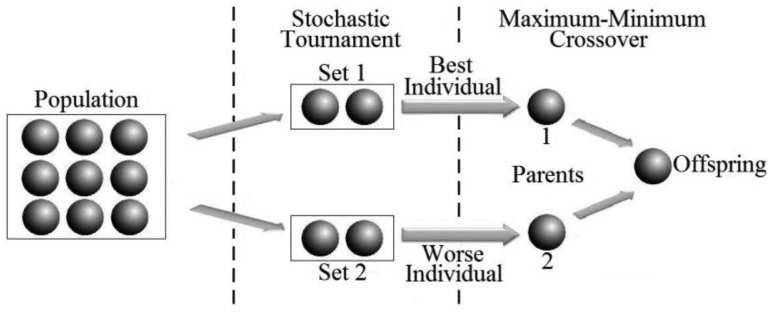
Selection and crossover in GA-MMC [14].

Furthermore, in PARs, the search spaces are vast. Thus, a crossover only with fittest individuals accelerates the convergence, but it requires high mutation rates to maintain the genetic diversity and global search, which increases processing time. Therefore, a remarkable feature of the developed GA is the new genetic operator Maximum-Minimum Crossover (MMC), which combines both the most and least fit individuals to increase genetic diversity. In Figure 10, there is an illustration of selection and crossover in GA-MMC. To control this process, a crossover rate, *T_C_*, is used, that determines whether the crossover is applied between a pair of individuals. The tests, taking into account the fitness value fitness, indicated a fixed rate *T_C_* = 35%.

In GA-MMC tests, each situation is simulated 10 times [55] to verify the ability of convergence and repeatability of GA results, given it is a probabilistic method. Therefore, simulations for controlling the main lobe tilt and maximizing RSLL were performed 10 times with conventional crossover, and another 10 times with MMC. In both cases, there was a success rate equal to or greater than 80% in tilt angle. However, GA-MMC allowed an average improvement of 1.6 dB in RSLL and of 20.9% in fitness. Therefore, MMC conducts a better search space scanning, avoiding premature convergence and high mutation rates.

#### 3.2.7. Mutation

The mutation genetic operator performs a global search within the space of solutions, randomly changing the genes value of the chromosome of a preselected individual. One way to maintain genetic diversity is to increase the probability of mutation, though it can also generate a purely random and time-consuming search. On the other hand, a high probability of crossover accelerates convergence, but can consequently result in an localized and ineffective search on multimodal problems, such as PARs. Thus, there must be a balance between crossover and mutation, which is called exploration–exploitation balance [16].

The real coding adopted in GA-MMC facilitates the process of mutation, which is done in a similar way to the crossover. It combines two individuals—one obtained from the population by a selection process of the fittest, and the other is freely generated, as in the initialization process, enabling global scanning. As in the crossover, mutation is controlled by a rate, *T_M_*, defined by the user, which usually has high values for real coding GAs [21,58]. However, with MMC, there is a better scanning of the search space, reducing *T_M_* and processing time, so that the tests indicated a fixed rate *T_M_* = 10%.

#### 3.2.8. Replacement

Genetic operators produce new individuals to be inserted into the population by the replacement process. In GA-MMC, substitution model uses the Steady State Genetic Algorithm, SSGA, an intermediate situation with faster convergence, when compared to the Replacement Genetic Algorithm, RGA (total replacement of the population), and the Simple Genetic Algorithm, SGA (substitution of only one or two individuals) [13]. In this case, elitism technique is used to reduce sampling errors, because it ensures the permanence of the best individuals from one generation to another, replacing only those with the worst fitness by individuals obtained from reproduction [16]. Thus, at the end of the evaluation with the objective function, the population is sorted by fitness value, and individuals below a cut line are replaced, resulting in a SSGA-45% (*T_C_* + *T_M_* = 45%).

#### 3.2.9. Termination Conditions

**Figure 11.**
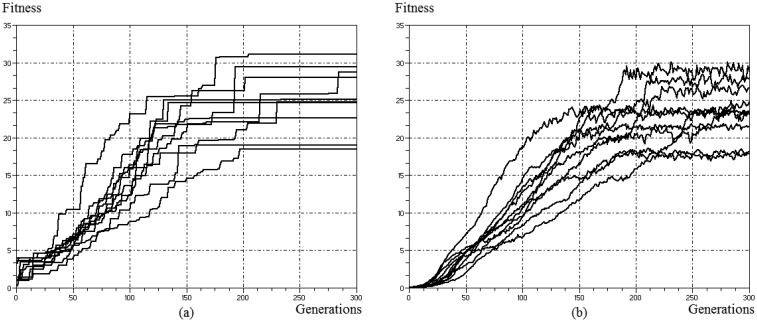
Evolution of population fitness in GA-MMC for 10 simulations (tilt angle equal to 30°) [13]. (**a**) Maximum Fitness. (**b**) Average Fitness.

The above processes are repeated iteratively so that the average quality of individuals increases at each cycle until the optimal solution is found. However, the result can be a set of optimal solutions (instead of one) or the optimum global value may be unknown, so that a termination condition should be adopted. In GA-MCC, considering the available time and processing capacity, tests indicated that the termination condition should be the maximum number of 300 generations, since higher values bring little improvement and increased processing time. In Figure 11, the evolution of the maximum and average fitness of the population in GA-MMC is shown throughout 10 simulations (tilt angle equal to 30° [13]). This confirms the ability of convergence and repeatability of results of GA and shows that the average fitness follows the maximum behavior after a few generations.

## 4. Results and Discussion

### 4.1. Preliminaries

The modeling step is the most difficult in the control design of the radiation pattern of phased array radars (PARs), because it involves an optimization problem with various restrictions, requiring the use of computing tools, such as genetic algorithms (GAs). In this context, Genetic Algorithm with Maximum-Minimum Crossover (GA-MMC) was developed to calculate offline the optimum values for excitation signals of the phased array (PA) elements, which are used to control the radiation pattern. GA-MMC is quite generic and flexible, requiring only changes in input parameters and the objective function to suit various applications. The algorithm was developed in Scilab software, since it is an easy-to-use computing environment and it allows creating functions on instruction files compatible with different hardware and operating systems [67]. The tests were run on multiple computers, taking advantage of Scilab portability. Each GA-MMC test lasted for a reasonably short amount of time on a PC with processor Intel Pentium 4 CPU 2.8 GHz, but with the use of most modern PCs, this processing time is reduced by about 60%. In addition, tests indicate that the use of a programming language, instead of Scilab, drastically decreases the processing time.

To test GA-MMC validity, a linear array was used, mainly because it represents a more challenging task for the optimization method, since it has less freedom to format the radiation pattern than a planar array does. Hence, a linear setting requires a rigorous control from the elements excitation signals. Moreover, as the GA-MMC is generic and allows adaptation to any type of element, isotropic radiators were chosen, for they reduce calculations during tests, and can be easily extended to other types of elements, such as dipoles or microstrip antennas [7]. They also allow a comparison with other studies [20,22].

The tests included two applications, in which the first one has two optimization objectives (optimize tilt and RSLL), and the second one is a continuation of the first, with three optimization objectives (optimize tilt, RSLL and null). In both applications, a PAR with similar structure to that outlined in Figure 7b is used. In this case, the angle of interest for the tilt varies from 0°–45°, since each face covers a 90° scanning area. Nevertheless, to test GA-MMC, tilt angles between 0° and 90° (180° scanning area) were considered.

In each test, 10 simulations were performed to verify the ability of convergence and repeatability of GA results [55]. The algorithm was considered approved on a test when converged in all simulations and reached operating conditions in at least 70% of the simulations. This percentage is used because GA has several probabilistic operators, progressing to an optimal solution by different paths each time it runs. Hence, tests should be analyzed in terms of success of the average or most of the results [22].

### 4.2. First Application: Tilt and Sidelodes Control

#### 4.2.1. Operation Conditions

**Figure 12.**
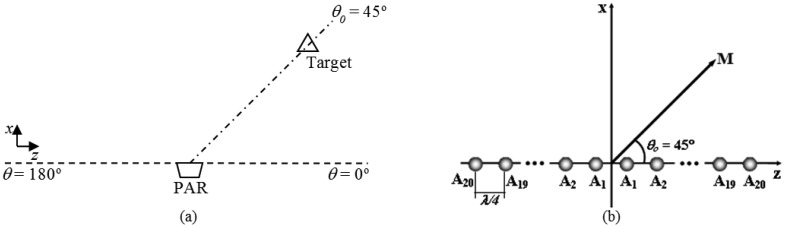
(**a**) PAR target tracking in θ_0_ = 45°. (**b**) PA used in the first application of GA-MMC tests, with θ_0_ = 45° [13].

In the first application, the objectives are to position the main lobe of radiation in the desired direction of the elevation plane or E-plane (to determine the tilt angle, θ_0_) and reduce the level of sidelobes (increase RSLL) as the objective function given in Equation (5). Then, GA-MMC determines the optimum value of the amplitudes (*A_n_* between 0 and 7, with increment equal to 1) and progressive phase (β between –π and +π, with increment 0.01 rad) for a linear PA excitation. This linear PA possess 40 isotropic elements (*N* = 2*M* = 40), symmetrically distributed, and an uniform spacing of a quarter wavelength (*d* = λ/4). To illustrate, Figure 12 contains an operating condition in which a PAR tracks a target in θ_0_ = 45° and details the associated linear PA used in the first GA-MMC application tests [13].

To analyze the GA-MMC effectiveness in the E-plane, tests with five directions of θ_0_ (90°, 60°, 45°, 30° and 10°) were conducted, which correspond to the main operation angles. The criterion adopted for considering the simulation results approved were: (i) maximum error of ±0.5° for the θ_0_, due to the resolution available (Δθ = 0.573° or 0.01 rad), (ii) RSLL ≥ 30 dB, for θ_0_ = 90° (broadside), (iii) RSLL ≥ 27 dB (3 dB less than broadside) to θ_0_ < 90°. As can be seen in (iii), major changes of the radiation pattern are required. These RSLL values are used only to test GA-MMC, because they are very high if compared to common values of parabolic reflector radars (PRRs) in rocket tracking systems (RTS), which have typical RSLL of 15–20 dB.

#### 4.2.2. Analysis of the Simulations

The results of 10 simulations for each desired tilt angle, θ_0_, are given in Table 1, which contains the number of tilt errors, NE_θ_, the average value of the calculated tilt angles, θ_C,Ave_, the number of RSLL errors, NE_RSLL_, the average value of RSLL, RSLL_Ave_, and the maximum value of RSLL, RSLL_Max_ [13]. The test results were satisfactory, since all simulations converged properly (according to Section 3.2.9), showing no tilt errors (NE_θ_ = 0% in all cases) and the desired RSLL was achieved in almost all simulations.

Thus, the desirable operating conditions were met in more than 90% of the tests, far greater than the minimum established (70% according to Section 4.1), validating the GA-MMC effectiveness. This indicates an excellent result for a probabilistic method. On the other hand, for the region of interest of a PAR with four faces (Figure 7b), θ_0_ ≥ 45°, the results would be even better, meeting the operating conditions in more than 97% of simulations of the tests total.

The RSLL has high values (RSLL_Max_ between 30.2 and 34.7 dB), with little difference between RSLL_Ave_ and RSLL_Max_, indicating stability in results. As expected, the largest number of errors occurred at RSLL with low values of θ_0_ (10° and 30°), near endfire condition, since it is very difficult to control the radiation pattern under such circumstance. In these cases, values for RSLL could be higher, but the objective function prioritizes θ_0_, due to the importance of tilt angle in PARs. This reduces the chance of getting even higher RSLL values, but it ensures achieving the operating requirements throughout the E-plane.

**Figure 13.**
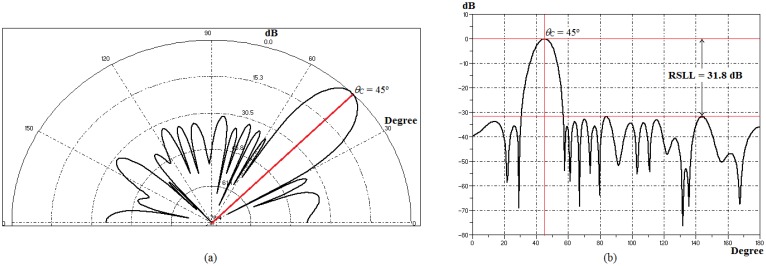
Radiation pattern for θ° = 45° in Table 2. (**a**) Polar form. (**b**) Cartesian form.

Table 2 contains the values of *A_n_* and β for individuals with best fitness obtained in each test simulations [13]. As each individual in the population contains only one side of PA (*M* = 20 elements), due to the amplitude symmetrical distribution, one notes that the value of *A_n_* is higher in the core elements, *A*_1_ to *A*_10_, and the absolute value of β increases as θ*_0_* decreases. Thus, GA-MMC reduced the value of *A_n_* in the side elements, in order to raise RSLL, while increased β to decrease θ_0_ (90°–10°). This was expected since β usually controls the main lobe tilt angle and *A_n_* controls the sidelobes level [7,13]. To illustrate, Figure 13 contains the polar and Cartesian forms in the E-plane of the radiation pattern optimized by GA-MMC for θ_0_ = 45° in Table 2.

In order to validate the optimization performed by GA-MMC, the simulation results are compared with data from references. In the first reference, there is a linear array of 40 isotropic elements symmetrically distributed, aiming at optimizing RSLL and θ_0_, obtaining RSLL of 32.9 dB to θ_0_ = 60°. Now, there is a GA with 200 individuals and binary coding of eight levels to *A_n_* and 216 levels to β [20]. In the second reference, there is a linear array of 40 isotropic elements symmetrically distributed, whose optimization objective is to increase RSLL, achieving results on the order of 20 dB to θ_0_ = 90°. This setting used a GA with 100 individuals, binary coding and two levels for *A_n_* [22].

Even though each of those references has their own peculiarities [20,22], their results confirm the success of the proposed optimization method. As comparison, GA-MMC reached RSLL = 34.7 dB, to θ_0_ = 90°, and RSLL = 32.6 dB, to θ_0_ = 60° (Table 1), values that are comparable or even larger than those cited in the references.

Additionally, GA-MMC is designed to meet many applications, including simultaneous optimization objectives. Therefore, *K_M_* = 1 was adopted, but it is noteworthy that, for *K_M_* = 0.05, GA-MMC provided RSLL > 37.0 dB, which is higher than that calculated by the deterministic Dolph-Tschebyscheff method (RSLL = 36.1 dB).

### 4.3. Second Application: Control of Tilt, Sidelobes and Nulls

#### 4.3.1. Operation Conditions

A classic application of PAs radiation pattern control is the direction of arrival (DoA) problem, which aims at separating multiple signals on the same frequency channel through spatial filtering algorithms and adaptive antennas. A DoA problem occurs when interfering signals of high power hinder or even prevent the reception of the desired signal, so that the signal-to-noise ratio is degraded or the input level of the receptor is saturated [13]. It is possible to reject such interference, positioning nulls of the radiation pattern in the directions of arrival of the interfering signals. As in the case of tilt angle control (first application), positioning these nulls requires control of the PAR elements excitation. This changes the radiation pattern, increasing the beamwidth and reducing gain and RSLL [13].

Then, in the second application of GA-MMC, there were three optimization objectives. The first two objectives are the same as the first application, which is to determine the main lobe tilt angle and reduce the sidelobes level. The third objective is to direct nulls of the radiation pattern to reject two interfering signals (e.g., jammers), *A*_I1_ and *A*_I2_, with known directions of arrival, according to the objective function given in Equation (6). The null is defined as the place of the radiation pattern with negligible transmission or reception capacity. In this paper, the adopted null value was 50 dB below the main lobe.

**Figure 14.**
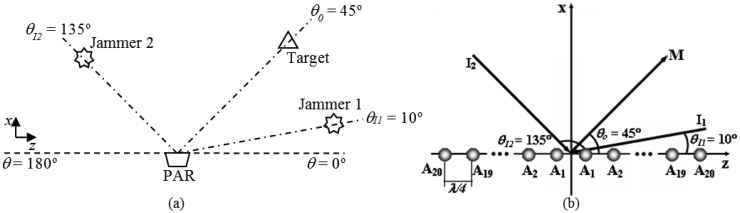
(**a**) PAR target tracking in θ*_0_* = 45° and jammers in θ_I1_ = 10° and θ_I2_ = 135°. (**b**) PA used in the tests of the second GA-MMC application, with θ_0_ = 45°, θ_I1_ = 10° and θ_I2_ = 135° [13].

GA-MCC uses the exact same operating conditions (*A_n_*, β, *N* e *d*) established for the first application. Furthermore, there are known directions of arrival, θ_I1_ and θ_I2_, for two simultaneous interfering signals. To illustrate, Figure 14 contains an operating condition in which the PAR tracks a target in θ_0_ = 45° and suffers interference from two jammers in θ_I1_ = 10° and θ_I2_ = 135°, as well as details the associated linear PA, which was used in the second GA-MMC application tests [13].

Due to the large number of possible operational situations, obtained by combining the values of θ_0_, θ_I1_ and θ_I2_, it was decided to test only the region of interest to a PAR of four faces (Figure 7b), that is, 45° ≤ θ_0_ ≤ 90°. Despite this simplification, it is possible to use GA-MMC in any operational situation even to check impossible situations in practice (as θ_0_ ≅ θ_I1_, for instance). Hence, to analyze the effectiveness of GA-MMC in the region of interest of the E-plane, tests for main operating conditions were chosen, as shown in Table 3. The adopted criteria for considering the simulation results approved were (i) maximum error of ±0.5° for the θ_0_, due to the resolution available (Δθ = 0.573° or 0.01 rad), (ii) signal-to-interference ratio, *SIR*, between main lobe (*A_M_*) and interference (maximum between *A_I1_* and *A*_I2_) greater than or equal to 50 dB, and (iii) RSLL ≥ 15 dB in all different directions from those of the main lobe and of the nulls. This RSLL range was adopted because it is at a typical value in PRRs for an RTS and due to the additional objective of the *SIR*.

#### 4.3.2. Analysis of the Simulations

The results of 10 simulations for each operating condition are summarized in Table 4, which, together with parameters in Table 1, contains the number of *SIR* errors, NE_SIR_, the average value of *SIR*, SIR_Ave_, and the maximum value of *SIR*, SIR_Max_ [13]. Each simulation only registered the worst *SIR* case, that is, the smallest null value for the two interferences. The overall result was satisfactory, since all simulations converged properly (according to Section 3.2.9) and there was only one tilt error in 80 simulations, no RSLL errors (NE_RSLL_ = 0% in all cases) and most of the tests showed no *SIR* error (NE_SIR_ = 0% in seven of the eight tests).

Hence, the operating conditions were met in 92% of simulations of the tests' total, indicating promising results for a probabilistic method and far greater than the minimum established (70% according to Section 4.1) to validate the GA-MMC effectiveness. As occurred in Section 4.2.2, RSLL showed high values (RSLL_Max_ between 22.2 and 29.6 dB) compared with parabolic reflector radars (PRRs) typically used in rocket tracking systems (RTS), with a typical RSLL range 15–20 dB.

Furthermore, there was little difference between RSLL_Ave_ and RSLL_Max_, which shows stability in results, considering the difficulty of the application with rigorous tilt and null restrictions. The sidelobes level could still be lower, but the objective function prioritizes θ_0_, θ_I1_ and θ_I2_, so it reduces the chance of increasing more RSLL. With respect to the *SIR*, very high values of the nulls of the radiation pattern were obtained in all tests (SIR_Max_ between 55.1 and 77.0 dB).

The greatest difficulty occurred in test 06 (θ_0_ = 45°, θ_I1_ = 55° and θ_I2_ = 35°), because, in this case, control leads to high deformation of radiation pattern below the main lobe, θ_0_ < 45°. This reduces null availability in this region and, consequently, makes it very difficult to eliminate an interfering signal only 10° below the main lobe (θ_I2_ = 35°). Even so, GA-MMC got an optimum solution in 40% of simulations.

**Figure 15.**
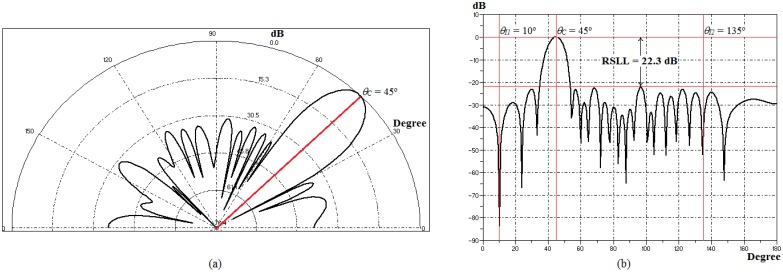
Radiation pattern for θ_0_ = 45°, θ_I1_ = 10° and θ_I2_ = 135° in Table 5. (**a**) Polar form. (**b**) Cartesian form.

In Table 5, the values of *A_n_* and β for individuals with best fitness obtained in each test simulations are presented [13]. As in Section 4.2.2, GA-MMC reduced the value of *A_n_* in the side elements, in order to raise RSLL, while increased β to decrease θ_0_ (90°–45°). This, once more, confirms that β controls the main lobe direction and *A_n_* controls the sidelobes level [7,13]. Regarding the *SIR*, several combinations of *A_n_* meet the null level specified for the application (50 dB), indicating the highly multimodal nature of the problem. GA-MMC searches one of these combinations, as long as they enable optimizing θ_0_ and RSLL. To illustrate, Figure 15 contains polar and Cartesian forms in the E-plane of the radiation pattern optimized by GA-MMC for θ_0_ = 45°, θ_I1_ = 10° and θ_I2_ = 135° in Table 5.

As it was done in Section 4.2.2, in order to validate the optimization performed by GA-MMC, simulation results are compared with data from references. Accordingly, there are similar examples with the proposed application, using GAs for the optimization of multiple objectives (such as null positioning) in controlling the radiation pattern of linear arrays with isotropic elements [53,54]. Once again, the results of these references demonstrate the GA-MCC success, since it shows null values comparable or even better than those obtained in previous works. In addition, there have been reports of difficulty in achieving nulls 40 dB below the main lobe [53]. On the other hand, considering 10 simulations for each operating condition tested, GA-MMC allowed values over 50 dB (peaking 77.0 dB), proving the repeatability and stability of results.

## 5. Conclusions/Outlook

The greatest difficulty in the design of phased array radars (PARs) used as sensors in a rocket tracking system (RTS) is the modeling step. It is a complex optimization problem due to the large number of possible solutions. In order to assist the design of PARs, the Genetic Algorithm with Maximum-Minimum Crossover (GA-MMC) optimization method was developed and tested. This method is used to optimize the electronic control of radiation pattern in phased arrays (PAs) and has a reconfigurable, multi-objective and with differentiated real coding GA, inspired by the physical structure of the array, in order to simplify the algorithm and decrease processing time. In addition, GA introduces the new genetic operator Maximum-Minimum Crossover (MMC), which has a different approach from conventional ones, because it performs crossover of the fittest individuals with the least fit individuals and, thereby, provides a better optimization in Pas' vast search space problems (around 10^20^ possibilities for the situations tested in this paper). This made it possible to increase the final population fitness by more than 20% and reduce processing time.

To test the method, common applications in radars were used, aiming at positioning the main lobe tilt, reducing the sidelobes level and directing nulls of the radiation pattern in the direction of arrival of interfering signals (e.g., jammers). Test results validated the GA-MMC, since operating conditions were met in more than 90% of the tests total in each proposed application. Hence, it was possible to obtain correct tilt angles and nulls, besides small sidelobes (30.2 dB ≤ RSLL ≤ 34.7 dB, for first application, and 22.2 dB ≤ RSLL ≤ 29.6 dB, for second application) and high values of nulls (55.1 dB ≤ SIR ≤ 77.0 dB). GA-MMC is also generic and reconfigurable for other applications, requiring only the need to inform new input parameters and/or the new objective function. Therefore, one promising outlook is to use GA-MMC in the design of PAs in a radar sensors network, in order to create a rocket tracking system (RTS) composed of phased array radars (PARs), for tracking soon after launch, and parabolic reflectors radars (PRRs), for tracking over long distances.

## Figures and Tables

**Table 1.** Test results of the first application of GA-MMC [13].

**θ_0_**	**NE_θ_**	**θ_C,Ave_**	**NE_RSLL_**	**RSLL_Ave_**	**RSLL_Max_**
**90**°	0%	89.9°	0%	31.9 dB	34.7 dB
**60**°	0%	60.2°	10%	30.9 dB	32.6 dB
**45**°	0%	45.2°	0%	29.6 dB	31.8 dB
**30**°	0%	29.8°	20%	28.9 dB	30.2 dB
**10**°	0%	9.7°	20%	28.9 dB	31.7 dB

**Table 2.** Individuals with higher fitness in tests of the GA-MMC first application [13].

**θ_0_**	**[*A****_1_* ***A****_2_* ***A****_3_* ***A****_4_* ***A****_5_* ***A****_6_* ***A****_7_* ***A****_8_* ***A****_9_* ***A****_10_* ***A****_11_* ***A****_12_* ***A****_13_* ***A****_14_* ***A****_15_* ***A****_16_* ***A****_17_* ***A****_18_* ***A****_19_* ***A****_20_***]**	**β**
**90°**	*[6 5 6 4 6 4 4 5 5 0 6 3 0 4 2 0 2 1 0 1]*	–0.04°
**60°**	*[7 7 7 7 6 6 6 5 6 4 5 3 4 3 2 3 1 3 0 2]*	–22.35°
**45°**	*[6 6 7 5 7 4 6 4 5 4 3 4 2 3 1 3 1 2 0 1]*	–31.63°
**30°**	*[6 6 6 6 6 6 5 5 4 4 3 4 2 3 2 2 1 1 1 1]*	–39.00°
**10°**	*[6 6 6 6 6 6 5 5 4 4 4 3 3 2 2 2 1 1 1 1]*	–44.31°

**Table 3.** Tests of the second application of GA-MMC [13].

**Test**	**θ_0_, θ_I1_ e θ_I2_**
**01**	90°, 10° e 170°
**02**	45°, 10° e 170°
**03**	90°, 130° e 140°
**04**	45°, 130° e 140°
**05**	90°, 100° e 80°
**06**	45°, 55° e 35°
**07**	90°, 135° e 10°
**08**	45°, 10° e 135°

**Table 4.** Test results of the second application of GA-MMC [13].

**Test**	**NE_θ_**	**θ_C,Ave_**	**NE_RSLL_**	**RSLL_Ave_**	**RSLL_Max_**	**NE_SIR_**	**SIR_Ave_**	**SIR_Max_**
**01**	10%	89.9°	0%	22.5 dB	25.0 dB	0%	59.1 dB	65.9 dB
**02**	0%	45.1°	0%	20.7 dB	25.3 dB	0%	56.6 dB	77.0 dB
**03**	0%	89.9°	0%	22.5 dB	26.6 dB	0%	51.5 dB	56.7 dB
**04**	0%	45.1°	0%	19.7 dB	22.2 dB	0%	54.1 dB	61.1 dB
**05**	0%	89.9°	0%	25.1 dB	29.6 dB	0%	54.6 dB	73.4 dB
**06**	0%	45.3°	0%	19.8 dB	23.0 dB	60%	53.0 dB	55.4 dB
**07**	0%	89.9°	0%	21.8 dB	24.4 dB	0%	52.4 dB	56.4 dB
**08**	0%	45.1°	0%	19.2 dB	22.3 dB	0%	51.8 dB	55.1 dB

**Table 5.** Individuals with higher fitness in the second GA-MMC application test [13].

**Test**	**[ *A****_1_* ***A****_2_* ***A****_3_* ***A****_4_* ***A****_5_* ***A****_6_* ***A****_7_* ***A****_8_* ***A****_9_* ***A****_10_* ***A****_11_* ***A****_12_* ***A****_13_* ***A****_14_* ***A****_15_* ***A****_16_* ***A****_17_* ***A****_18_* ***A****_19_* ***A****_20_* **]**	**β**
**01**	*[6 7 5 7 1 7 4 4 6 5 1 5 4 1 5 0 3 1 1 1]*	–0.10°
**02**	*[6 6 6 4 7 4 7 4 6 3 6 1 5 2 4 0 2 2 2 1]*	–32.09°
**03**	*[6 6 7 5 7 7 4 4 5 6 2 5 4 3 3 2 3 2 1 2]*	0.03°
**04**	*[5 6 6 6 5 4 4 5 6 5 1 5 1 5 1 5 2 4 0 3]*	–32.01°
**05**	*[6 7 6 7 4 7 5 5 3 7 2 2 6 0 3 3 1 2 1 2]*	–0.25°
**06**	*[5 7 6 7 4 7 5 7 2 6 4 5 5 2 3 3 6 3 5 2]*	–31.53°
**07**	*[5 5 7 4 5 5 4 6 2 5 4 2 5 4 0 3 3 2 2 3]*	–0.19°
**08**	*[6 5 5 6 6 6 5 5 5 5 4 4 2 5 1 5 2 2 2 4]*	–31.80°
